# TNF receptor-associated factor 6 (TRAF6) plays crucial roles in multiple biological systems through polyubiquitination-mediated NF-κB activation

**DOI:** 10.2183/pjab.97.009

**Published:** 2021-04-09

**Authors:** Mizuki YAMAMOTO, Jin GOHDA, Taishin AKIYAMA, Jun-ichiro INOUE

**Affiliations:** *1 Research Center for Asian Infectious Diseases, The Institute of Medical Science, The University of Tokyo, Tokyo, Japan.; *2 Laboratory for Immune Homeostasis, RIKEN Center for Integrative Medical Sciences, Yokohama, Kanagawa, Japan.; *3 Research Platform Office, The Institute of Medical Science, The University of Tokyo, Tokyo, Japan.

**Keywords:** TRAF6, NF-κB, signal transduction, ubiquitin

## Abstract

NF-κB was first identified in 1986 as a B cell-specific transcription factor inducing immunoglobulin κ light chain expression. Subsequent studies revealed that NF-κB plays important roles in development, organogenesis, immunity, inflammation, and neurological functions by spatiotemporally regulating cell proliferation, differentiation, and apoptosis in several cell types. Furthermore, studies on the signal pathways that activate NF-κB led to the discovery of TRAF family proteins with E3 ubiquitin ligase activity, which function downstream of the receptor. This discovery led to the proposal of an entirely new signaling mechanism concept, wherein K63-ubiquitin chains act as a scaffold for the signaling complex to activate downstream kinases. This concept has revolutionized ubiquitin studies by revealing the importance of the nonproteolytic functions of ubiquitin not only in NF-κB signaling but also in a variety of other biological systems. TRAF6 is the most diverged among the TRAF family proteins, and our studies uncovered its notable physiological and pathological functions.

## Introduction

**Figure 1.**
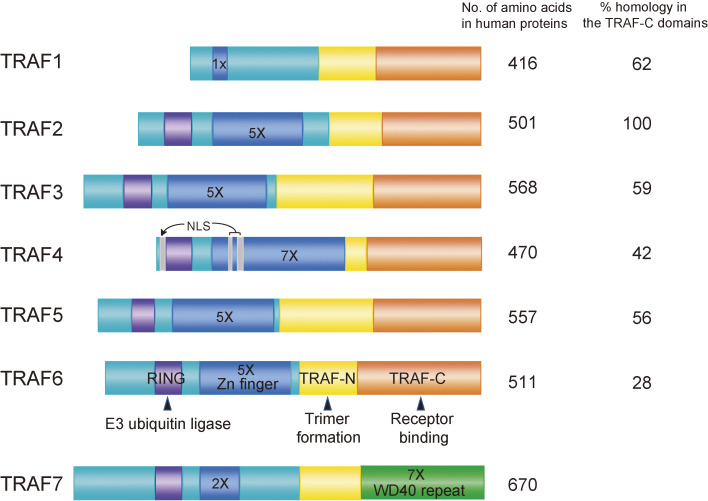
Structure of the TRAF family of proteins. The TRAF domain consists of TRAF-N and TRAF-C. The TRAF-N domain contains a coiled-coil structure and mediates trimer formation. The TRAF-C domain binds to upstream proteins such as the cytoplasmic tail of TNFRSF or IRAK. Although TRAF7 has a TRAF-N domain, it has seven WD40 repeats instead of a TRAF-C domain. TRAF2, TRAF3, TRAF5, and TRAF6 have five Zinc fingers (5×), whereas TRAF1, TRAF4, and TRAF7 have one (1×), seven (7×), and two (2×) Zinc fingers, respectively. TRAF4 has two potential nuclear localization signals (NLSs). Homologies in the TRAF-C domains are shown when each TRAF-C domain is compared with that of TRAF2.

**Figure 2.**
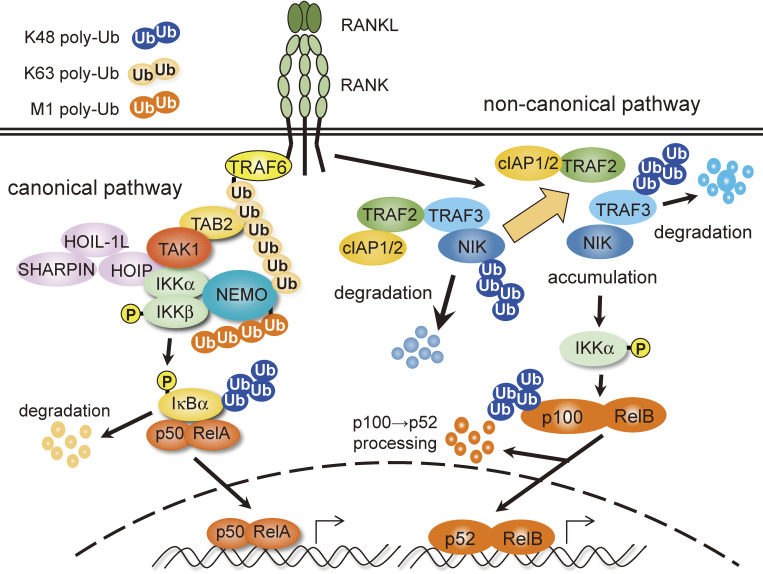
Canonical and non-canonical NF-κB pathways. Canonical and non-canonical NF-κB pathways emanating from RANK are illustrated. In the canonical pathway, the K63-Ub chain generated by TRAF6 acts as a platform to form a signal complex consisting of TAK1/TAB2, IKKα/IKKβ/NEMO (IKK complex) and HOIP/HOIL-1L/SHARPIN (linear ubiquitin chain assembly complex [LUBAC]) to activate the IKK complex. IKK then phosphorylates IκBα to target IκBα for K48 polyubiquitination, which leads to the degradation of ubiquitinated IκBα by the proteasome, allowing the p50/RelA complex to enter the nucleus and activate target genes. In the non-canonical pathway, under unstimulated conditions, NIK is persistently degraded by the proteasome due to K48 polyubiquitination by cIAP1/2 because the TRAF2/TRAF3 heterodimer acts as a molecular bridge between NIK and cIAP1/2. Stimulation that activates the non-canonical pathway induces stabilization and activation of NIK, occasionally with a concomitant degradation of TRAF2 and TRAF3. This pathway involves activation of the IKKα homodimer, which then phosphorylates the C-terminal domain of p100 and targets it for proteasome-dependent processing to generate p52, allowing the p52/RelB complex to enter the nucleus and activate target genes.

Although members of the tumor necrosis factor (TNF) receptor superfamily (TNFRSF) are crucial in inflammation, the immune system, and organ development and are involved in the onset and development of various diseases,^1)^ little was known about their signal transduction mechanisms because no catalytic activity or typical functional amino acid motifs had been found in their cytoplasmic tails. However, the development of protein purification techniques and gene identification based on protein–protein interactions, such as the yeast two-hybrid system, led to the discovery of the TNF receptor-associated factor (TRAF) family of proteins, which directly bind to the cytoplasmic tail of TNFRSF upon ligand stimulation. To date, seven members of the TRAF family, TRAF1–7, have been identified (Fig. 1).^2–12)^ Among them, TRAF2, 5, and 6 activate nuclear factor-κB (NF-κB) and activator protein 1 (AP-1) when overexpressed. Accumulating evidence indicates that TRAF2, 5, and 6 are involved in the canonical NF-κB pathway, while TRAF2 and TRAF3 are involved in the non-canonical NF-κB pathway (Fig. 2).^13)^ Since TNFRSF-induced canonical and non-canonical NF-κB activation spatiotemporally cooperate to control multiple biological processes,^14)^ the role of each TRAF protein and the molecular mechanism of its activation needed to be clarified. In this review, a brief overview of the TRAF family is provided in the first section. We then focus on TRAF6, which we have investigated extensively, in the subsequent sections.

## Our discovery of TRAF: a story of fierce competition

Our starting point was CD40, a member of the TNFRSF, which is expressed in antigen-presenting cells such as B cells, dendritic cells, and macrophages and is essential for the activation of these cells by helper T cells expressing CD40 ligand.^15)^ CD40 is central to the immune response, playing an important role in clonal selection, immunoglobulin class switching, immune tolerance, germinal center formation, and T cell-dependent antibody production. We started this research in the hope of elucidating the signaling in these important processes at the molecular level. Because the cytoplasmic tail of CD40 does not contain any structures that would predict its enzymatic activity, we thought that some other protein might bind to the cytoplasmic region. Therefore, we began investigating the mechanism of CD40 signaling using a yeast two-hybrid system,^16)^ which was becoming popular at that time. During our screening, Rothe *et al.* in Goeddel’s group in Genentech reported TRAF1 and TRAF2, which are TNFR type II signaling factors.^2)^ Soon after, Cheng *et al.* in Baltimore’s group in MIT reported CD40 receptor-associated factor 1 (CRAF1; later TRAF3) as a CD40 signaling factor in the same way as ours,^3)^ and Régnier *et al.* in Rio’s group in France reported TRAF4 as a gene strongly expressed in breast cancer.^7)^ Our screening program started to take off at that time, and we identified two novel TRAF-like proteins, which we first called CRAF2 and CRAF3, because we used the CD40 cytoplasmic tail as bait for the yeast two-hybrid screening.^9,12)^ However, upon submitting our CRAF2 paper, the editor informed us that the same molecule had been identified as TRAF5 in another journal, and the paper was already in press. Therefore, we changed the name of CRAF2 to TRAF5 when published.^9)^ Due to this change, we also renamed CRAF3 to TRAF6. Furthermore, soon after we submitted our TRAF6 paper, Cao *et al.* in Goeddel’s group published a paper on identifying TRAF6 from the expressed sequence tag (EST) database and reported it as a signaling factor of the interleukin-1 receptor (IL-1R).^11)^ Our paper on TRAF6 was also accepted soon after.^12)^

## What is the TRAF family?

Apart from TRAF7, all other TRAF proteins share a common structure at the C-terminus called the TRAF-C domain (Fig. 1).^17)^ The TRAF domain consists of two domains, TRAF-C and TRAF-N. TRAF-C is highly conserved within the TRAF family but is unique to TRAF and is involved in the binding of TRAF to upstream receptors. On the other hand, TRAF-N, which is relatively poorly conserved, adopts a coiled-coil structure and is involved in TRAF trimer formation. The TRAF trimer is recruited to the cytoplasmic region of TNFSFR, another trimer-forming protein, in a ligand-stimulated manner. In TRAF7, there is no TRAF-C domain, and the corresponding site consists of seven WD40 repeats.^18)^ Because the WD40 repeat is a motif responsible for protein–protein interactions as well as the TRAF-C domain itself, it is possible that TRAF7 functions via a mechanism similar to that of the other six TRAFs. It has been shown recently that heterozygous missense variants in TRAF7 lead to combined developmental delays and congenital malformation called TRAF7 syndrome,^19,20)^ indicating that TRAF7 is crucial for development. However, the authors questioned whether TRAF7 should be added to the TRAF family because it lacks the TRAF-C domain. TRAF1 through TRAF6 were cloned over a 2-year period from 1994 to 1996, and TRAF7 was cloned 8 years later. Therefore, after the cloning TRAF5 and TRAF6 cDNAs, we focused our attention on TRAF6 because its TRAF-C domain is the most diverged among the six TRAFs (Fig. 1). In fact, the TRAF-C domain of TRAF6 recognizes amino acid sequences that are different from those recognized by other TRAFs. The consensus binding site for TRAF6 is Pro-X-Glu-X-X-Acidic/Aromatic,^21,22)^ whereas that for TRAF2, TRAF3, and TRAF5 is Pro-X-Gln-X-Thr.^21,23,24)^ Therefore, we hypothesized that TRAF6 had unique biological and pathological functions different from those of other TRAFs. As described below, the generation of TRAF6-deficient mice revealed that TRAF6 plays crucial roles in several critical biological processes, and that loss of TRAF6 cannot be compensated for by other TRAFs.^25,26)^

## Essential roles of TRAF6 in biological systems

In physiological conditions, is TRAF6 involved in NF-κB activation? If so, what biological phenomena involve TRAF6? To answer these questions, we generated TRAF6-deficient (TRAF6-KO) mice. TRAF6-KO mice were born in smaller numbers than the expected Mendelian ratio, and they died within 2 weeks of birth.^25)^ These findings strongly suggested that TRAF6 is involved in physiologically important biological processes. In the following sections, we describe the physiological importance of TRAF6, as revealed by our analysis of TRAF6-KO mice.

### 1 Osteopetrosis — abnormal osteoclast differentiation (Fig. 3).

Based on previous studies, we suspected bone abnormalities in TRAF6-KO mice, because of tooth eruption failure, as assessed by gross observation. Subsequent X-ray CT analysis revealed that the bone marrow cavity was filled with trabecular bone, indicating that TRAF6-KO mice display osteopetrosis.^25,26)^ Further pathological analysis revealed that osteoclasts, which resorb bone, were hardly formed. Osteoclasts are differentiated in response to stimulation of the receptor activator of NF-κB (RANK) on monocyte-derived osteoclast progenitor cells with the RANK ligand (RANKL).^27)^ RANK is a member of the TNFRSF that activates NF-κB and AP-1. However, in TRAF6 deficiency, the signal downstream of RANK was blocked, and the activation of these transcription factors was markedly suppressed.^28)^ Later, we found three TRAF6 binding sites in the RANK cytoplasmic tail,^29)^ and showed that TRAF6 binds to these sequences in a RANKL stimulus-dependent manner and subsequently activates NF-κB and AP-1, which, together with calcineurin activation, in turn activates nuclear factor of activated T-cells, cytoplasmic 1 (NFATc1).^29)^ NFATc1 acts as a master transcription factor for osteoclastogenesis, inducing the gene expression required for osteoclast differentiation.^27)^ Therefore, TRAF6 is crucial for bone homeostasis.

### 2 Defective lymph node organogenesis (Fig. 4).

TRAF6-KO mice were defective in lymph node formation throughout the body,^25,26)^ because RANK in IL-7R-positive fetal lymphotoxin producer cells in the lymph node primordium cannot transmit signals to secrete LTα_1_β_2_ due to TRAF6 deficiency.^30)^ Lack of LTα_1_β_2_ secretion results in the inability of mesenchymal cells to secrete chemokines such as CCL19 and CCL21, which in turn prevents the recruitment of lymphocytes, constituting lymph nodes, into the primordium.

### 3 Autoimmune diseases due to defective T cell self-tolerance — abnormal differentiation of thymic medullary epithelial cells (Fig. 5).

In the thymus, cortical epithelial cells (cTECs), medullary epithelial cells (mTECs), and dendritic cells (DCs) are arranged in a three-dimensional structure to form a functional microenvironment.^31,32)^ T cells differentiate and mature in this microenvironment to learn self and non-self by deleting autoreactive T cells, which strongly interact with self-antigens. This is called negative selection, and it is an important function of the thymus to prevent autoimmune reactions.^32)^ In mTECs, many proteins (tissue-specific antigens [TSAs]) that normally function in peripheral tissues are ectopically expressed (promiscuous gene expression), and when presented with the MHC, T cells that react with these autoantigens are eliminated via apoptosis.^33)^ In the thymus of TRAF6-KO mice, the differentiation and maturation of mTECs were insufficient, and their spatial arrangement in the thymus was abnormal. Autoimmune regulator (Aire) expression, involved in the expression of several TSAs, was markedly reduced compared with the wild-type (WT), as was TSA expression itself.^34)^ On the other hand, the lungs, liver, pancreas, and kidneys of TRAF6-KO mice showed autoimmune-like inflammation and autoantibodies that reacted with these tissues were present in serum of TRAF6-KO mice, indicating the development of autoimmune diseases.^34)^ Furthermore, when the thymic stroma from WT or TRAF6-KO fetuses was transplanted into the kidney capsule of thymus-free nude mice, T cells differentiated normally in mice receiving the WT thymic stroma, but the TRAF6-KO thymus transplanted mice showed an autoimmune-like inflammation and autoantibody production. These results clearly indicated that the thymic abnormality caused by TRAF6 deficiency is the cause of autoimmune disease. In other words, TRAF6 signaling in mTEC progenitors somehow upregulates Aire and TSA expression, completes the differentiation and maturation of mTECs, and establishes negative selection of T cells in the thymus, thereby preventing the development of autoimmune responses. So, what is the TRAF6 signal at work here? After further analysis, we proposed that RANK stimulation on mTEC progenitors with RANKL on T cells activates the canonical NF-κB pathway to induce expression of the transcription factor RelB, which heterodimerizes with p100. RANK stimulation also activates the non-canonical NF-κB pathway to induce accumulation of NF-κB-inducing kinase (NIK) leading to the proteolytic processing of p100 to p52. The resulting p52/RelB heterodimer then induces the expression of genes required for mTEC differentiation.^35,36)^ Indeed, an NF-κB binding element is present in a conserved non-coding sequence upstream of Aire genes, suggesting that RANK-induced p52/RelB promotes Aire expression.^37,38)^ Aire then promotes TSA expression to generate an adequate thymic microenvironment to establish T cells’ negative selection.

### 4 Decreased mammary epithelial stem cell numbers and defective expansion of pregnancy-induced mammary epithelial cells (Fig. 6).

The normal mammary gland comprises two types of epithelial cells (luminal epithelial cells and basal epithelial cells) differentiated from mammary epithelial stem cells, which develop duct-like lumens in the breast during puberty.^39)^ Additionally, during pregnancy, progesterone stimulation reactivates mammary epithelial stem cells, which, in addition to the ducts, develop pouch-like structures called lobules composed of luminal and basal epithelial cells in preparation for milk production after giving birth. Following weaning, the lobules undergo apoptosis and regress to their pre-pregnancy state.^40–42)^ Thus, mammary gland tissues are actively developing and retracting, even in adults, and their stem cell maintenance mechanisms are important for normal mammary gland development. Of note, mammary gland development is regulated by different signaling pathways during puberty and pregnancy. During pregnancy, progesterone stimulates the progesterone receptor on luminal epithelial cells to induce RANKL expression. RANKL then stimulates RANK on both luminal and basal epithelial cells to activate NF-κB,^43)^ AKR mouse thymoma (AKT, also known as protein kinase B (PKB))^44)^ and inhibitor of DNA binding 2 (Id2).^45)^ We found that TRAF6-dependent NF-κB activation is crucial for mammary gland development as follows.^43)^

TRAF6-deficient mammary glands, like RANK-deficient^44,46)^ and IκB kinase α (IKKα)-mutant^47)^ mammary glands, showed normal mammary gland development during puberty but severely impaired mammary gland development during pregnancy. We analyzed the frequency of mammary epithelial stem cells and luminal progenitor cells in pre-pregnant mammary tissue. We found that both types of undifferentiated cells were significantly reduced in TRAF6-deficient mammary glands, indicating that TRAF6 is crucial for maintaining undifferentiated mammary epithelial cells.^43)^ Furthermore, we analyzed the gene expression profiles of gestational luminal and basal epithelial cells and found that TRAF6-deficient mammary glands also induced sufficient expression of Casein and whey acidic proteins, necessary for milk production from the matured luminal cells. The suppression of these maturation markers in RANK-deficient mammary glands^44)^ suggests that RANK signaling required for maturation (TRAF6-independent Id2 pathway)^45)^ is activated normally in TRAF6-deficient mammary glands, whereas the signaling required for increasing the number of mammary epithelial cells is inhibited. We then investigated cell proliferation and cell death and found that the RANK-TRAF6-dependent cell proliferation signal requires AKT-mediated RB phosphorylation and that the RANK-TRAF6-dependent anti-apoptotic signal requires classical NF-κB activation to induce cIAP1/2 and A20 expression. Taken together, our results revealed that TRAF6 is involved in the maintenance of undifferentiated mammary epithelial cells such as stem cells and luminal progenitor cells in the pre-pregnant state and in cell proliferation and the survival of differentiated mammary epithelial cells by activating downstream of AKT and NF-κB signaling during pregnancy.

### 5 Hypohidrotic ectodermal dysplasia — defective formation of skin appendices (Fig. 7).

Hypohidrotic ectodermal dysplasia is the most common form of hereditary ectodermal dysplasia in humans.^48)^ It has three main features: anhidrosis (low sweat), sparse hair, and hypoplasia of the teeth. Sweat-related symptoms are due to the lack or hypoplasia of sweat glands. Consequently, body temperature regulation is impaired, and depressive symptoms and heat stroke repeatedly occur under high fever, leading to retardation of intellectual development and even death in infants. Loss of TRAF6 results in the failure of epidermal appendices such as guard hair follicles, sweat glands, sebaceous glands of the back skin, meibomian glands, anal glands, prostate glands, and other modified sebaceous glands.^49)^ Except for sebaceous gland defects, these abnormal phenotypes are identical to those observed in *tabby* (*Ta*), *downless* (*dl*), and *crinkle* (*cr*) mice, which are models of human hypohidrotic ectodermal dysplasia.^50–52)^ Expression of mucosal addressin cell adhesion molecule-1 (MADCAM-1), an early marker of hair follicle formation, was not observed in the skin of TRAF6-deficient fetuses.^49)^ Thus, TRAF6 is essential for the development of epidermal appendices. Although mutation of the dl protein/ectodysplasin receptor (EDAR) induces hypohidrotic ectodermal dysplasia, TRAF6 does not bind to the cytoplasmic region of EDAR. In contrast, TRAF6 binds to the X-linked ectodysplasin-A2 receptor (XEDAR) and the TNFRSF expressed in mice embryos (TROY).^49)^ Furthermore, TRAF6 is essential for XEDAR-mediated activation of NF-κB.^49)^ These results suggest that TRAF6 might be involved in the development of epidermal appendices by transducing signals emitted from XEDAR and TROY.

### 6 Defective Toll-like receptor (TLR)/IL-1R signaling and cytoplasmic RNA/DNA signaling (Figs. 8 and 9).

**Figure 8.**
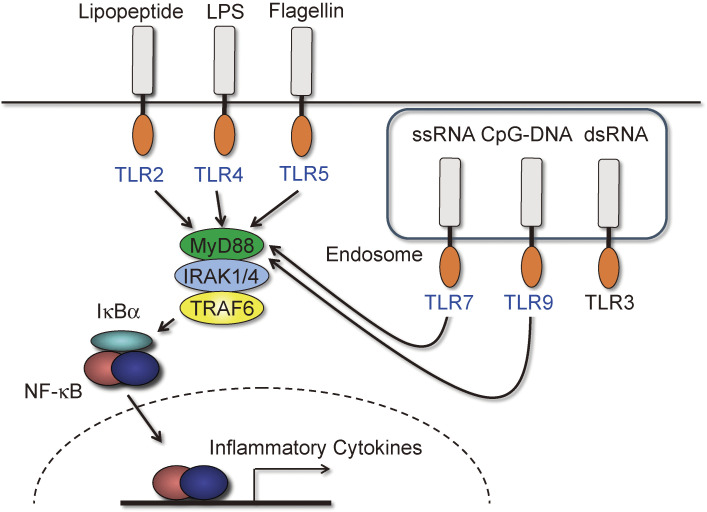
TRAF6 is involved in TLR signaling except for TLR3. TRAF6 is downstream of MyD88 in NF-κB activation during TLR signaling. Because the MyD88-TRAF6 axis is involved in TLR2, 4, 5, 7, and 9 signaling, NF-κB activation and cytokine production through these receptors are abolished in the absence of TRAF6. However, interferon production via these receptors is intact even in the absence of TRAF6. TRAF6 deficiency does not affect TLR3 signaling in terms of NF-κB activation and the production of cytokines and interferons.

The signaling pathway from TLRs is mediated by Toll/IL-1R (TIR) domain-containing adaptor molecules, and TRAF6 had been shown to activate NF-κB and MAPKs downstream of these TIR domain-containing proteins, inducing the production of pro-inflammatory cytokines.^53,54)^ However, the exact role of TRAF6 in signaling from individual TLRs was initially not adequately addressed. We analyzed macrophages from TRAF6-KO mice and made the following observations^55)^: 1) Ligands for TLR2, TLR5, TLR7, and TLR9 were unable to induce the activation of NF-κB and MAPKs or production of pro-inflammatory cytokines. 2) Cytokine production by ligands for TLR4 was markedly reduced. However, activation of NF-κB and MAPK was comparable to that of WT macrophages, albeit with a delay. These results indicate that TRAF6 is essential for MyD88-dependent signaling but not for TIR domain-containing adaptor-induced IFN-β (TRIF)-dependent signaling (Fig. 8).

**Figure 9.**
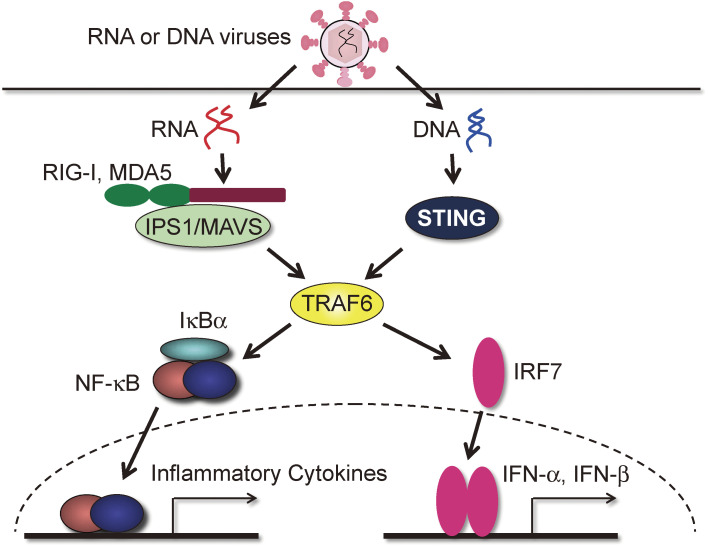
TRAF6 is involved in cytoplasmic RNA and DNA signaling. In the absence of TRAF6, NF-κB activation, cytoplasmic RNA, or DNA-induced NF-κB-induced cytokine production and interferon production were significantly inhibited. In RNA signaling, TRAF6 functions downstream of IPS1/MAVS. In DNA signaling, TRAF6 functions downstream of STING.

In addition to TLRs, cytoplasmic RNA sensors, RIG-like helicases (RLHs) are essential for antiviral responses.^56)^ However, the contribution of TRAF6 to the detection of cytosolic viral nucleic acids was controversial, and the involvement of TRAF6 in IRF activation had not been thoroughly investigated. We first showed that a lack of TRAF6 resulted in enhanced viral replication after RNA virus infection and a significant decrease in the production of IL-6 and type I IFN.^57)^ NF-κB and IRF7 activation were significantly impaired during RLH signaling in the absence of TRAF6, but not IRF3 activation. We also showed that TRAF6 deficiency impaired the cytoplasmic DNA-induced antiviral response and that this impairment was due to defective activation of NF-κB and IRF7.^57)^ Thus, TRAF6 mediates cytoplasmic viral DNA- and RNA-triggered antiviral responses in a manner distinct from TLR signaling (Fig. 9).

### 7 Defective NGF signaling in Schwann cells (Fig. 10).

Neurotrophin receptor p75 activation elicits conflicting cellular signals.^58)^ Depending on the cellular context, following neurotrophin stimulation, p75 either promotes survival or induces apoptosis of Schwann cells and principal glial cells of the peripheral nervous system. p75-mediated apoptosis occurs via JNK activation, whereas survival signaling is mediated by NF-κB activation. TRAF6 has been identified as an interactor of the p75 cytoplasmic tail; therefore, we addressed the role of TRAF6 in p75 signaling using TRAF6-KO Schwann cells in collaboration with Carter’s group in Vanderbilt University.^59)^ NGF activated NF-κB in WT Schwann cells while NF-κB activation was significantly reduced in the absence of TRAF6. Similarly, NGF did not activate JNK in TRAF6-KO Schwann cells. In addition, the lack of TRAF6 resulted in the failure of p75-mediated apoptosis. In sympathetic neurons derived from WT superior cervical ganglia (SCG), BDNF-binding p75 induced JNK activation and apoptosis, whereas it did not in the absence of TRAF6. Furthermore, *in vivo* spontaneous cell death in the SCG of postnatal day 4 TRAF6-KO mice was reduced by 55.6% compared with WT mice according to TUNEL-positive cells. These results indicate that TRAF6 is crucial in p75 signaling to induce apoptosis.

## Pathological roles of TRAF6 (Fig. 11)

**Figure 11.**
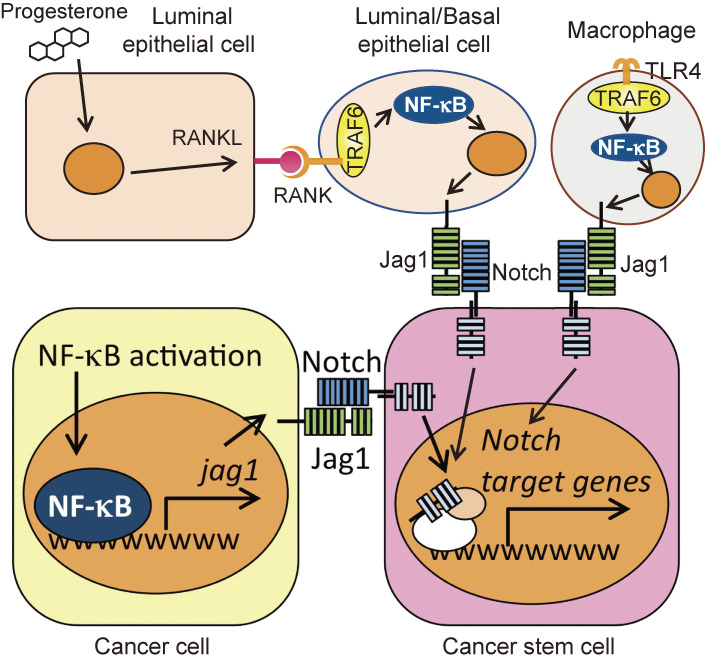
Possible involvement of the RANK-TRAF6-NF-κB axis in cancer stem cell maintenance in triple-negative breast cancer. Progesterone induces the expression of RANKL in luminal epithelial cells. RANKL simulates RANK on both basal and luminal epithelial cells to activate NF-κB via TRAF6. TLR4 stimulation by bacterial infection or endogenous TLR4 ligands also activates NF-κB via TRAF6. The resulting NF-κB activation induced the expression of JAG1, which then stimulated NOTCH on cancer stem cells to maintain or expand the stem cell population (upper half). Aberrant and constitutive NF-κB activation in basal subtype breast cancer induces expression of JAG1, which in turn stimulates NOTCH on cancer stem cells to maintain or expand the stem cell population (lower half).

Although NF-κB activation is tightly regulated in normal cells, it is constitutively activated in various cancers to induce cancer cell survival, proliferation, and metastasis.^60,61)^ We demonstrated that NF-κB is constitutively and strongly activated in triple-negative breast cancers (TNBCs), including basal-like and claudin-low breast cancer subtypes compared with luminal-like and erbB2-enriched subtypes and that NF-κB maintains cancer cell survival.^62–65)^ We also reported that basal-like breast cancer-specific NF-κB-JAG1-Notch signaling maintains breast cancer stem cells.^64)^ Several research groups had reported NF-κB activation in breast cancer stem cells prior to our analysis.^66,67)^ However, these reports suggested that NF-κB might be activated in breast cancer stem cells themselves to induce proliferation and survival, proposing a cell-autonomous role of NF-κB activation. In contrast, we focused on the activation of signal transduction via cell-cell interaction because the expression of various ligand molecules is induced downstream of NF-κB.^64)^ When basal subtype breast cancer cell line HCC1937 with high NF-κB activation induced by ectopic expression of constitutively active IKKβ (HCC1937^high^) was co-cultured with the same breast cancer cells with unaltered NF-κB activation (HCC1937^normal^) for 12 days, the percentage of cancer stem cells in HCC1937^normal^ became significantly higher than that in HCC1937^normal^ without co-culture. In this study, we found that the expression of Notch target genes was increased in HCC1937^normal^ after co-culture with HCC1937^high^. Based on these results, we focused on Notch signaling and found that in various basal-like breast cancer cell lines, JAG1, a Notch ligand, was induced by NF-κB activation. In other words, we found that NF-κB activation in cancer cells surrounding cancer stem cells induced the expression of JAG1 and activated Notch signaling in cancer stem cells, thereby creating a mechanism to maintain those cancer stem cells (Fig. 11, lower half).

As explained above, the RANK-TRAF6-NF-κB pathway is activated during pregnancy in normal mammary glands, leading to the proliferation and survival of mammary epithelial cells.^43)^ In addition, progesterone, which increases in concentration during pregnancy and the sexual cycle, strongly induces RANKL expression in luminal epithelial cells and stimulates RANK in both luminal and basal epithelial cells. This RANK stimulation not only induces cell proliferation and survival but also induces the expression of JAG1 (Fig. 11, upper half), which is likely to maintain breast cancer stem cells (Fig. 11). Other groups have reported that RANK signaling is also involved in breast cancer development by mechanisms distinct from ours.^68,69)^ Moreover, we demonstrated that TRAF6-mediated TLR4 signaling also induces JAG1 expression in macrophages. These data indicate that TRAF6 may play an important role in the progression of breast cancer through inflammation. Therefore, TRAF6 is likely to be involved in the physiological development of the mammary gland and, at the same time, contribute to breast cancer development.

As for the role of TRAF6 in carcinogenesis, it has been reported that the Ras-TRAF6-NF-κB pathway plays an important role in the oncogenesis of lung adenocarcinoma,^70)^ and that increased expression of TRAF6 is responsible for the development of the 5q^−^-type myelodysplastic syndrome.^71,72)^

## How does TRAF6 activate NF-κB? (Figs. 2 and 12)

Studies on the molecular mechanisms by which TRAF6 activates NF-κB have led to an innovative concept in signal transduction research. It began with Zhijian James Chen’s pioneering work published in 2000^73)^ & 2001.^74)^ Without stimulation, IκBα, an inhibitor of NF-κB, associates with NF-κB and masks the nuclear localization signal of NF-κB, thereby sequestering NF-κB in the cytoplasm. Upon stimulation, IκBα is phosphorylated by the IκB kinase (IKK) complex, which is composed of the catalytic subunits IKKα/β and the regulatory subunit NEMO. IκBα is subsequently modified with K48-type polyubiquitin chains (K48-Ub chains) and degraded by the proteasome, resulting in the nuclear translocation of NF-κB (Fig. 2, canonical pathway). Chen and colleagues found that TRAF6 acts as an E3 ubiquitin ligase to form K63-type polyubiquitin chains (K63-Ub chains) and activates the downstream kinase TAK1, which in turn activates IKKβ by phosphorylation.^73,74)^ Before his findings, many researchers considered polyubiquitination to lead only to protein degradation. Chen was the first to propose that K63-Ub chains do not induce protein degradation but rather serve as a scaffold for forming signal complexes with multiple proteins. More specifically, TAK1 associated with TAB2 accumulates on K63-Ub chains via TAB2 because TAB2 can bind to K63-Ub chains. This TAK1 accumulation results in the intermolecular self-phosphorylation of TAK1, leading to TAK1 activation. TAK1, in turn, phosphorylates IKKβ associated with K63-Ub chains via NEMO because NEMO can bind to the K63-Ub chains, thereby allowing TAK1 to phosphorylate IKKβ and activate the IKK complex. This concept regarding ubiquitination-mediated activation of kinases, which was previously unreported, reaffirmed the importance of post-translational modifications and significantly advanced our understanding of ubiquitin’s multi-faceted roles. Since then, his concept has been confirmed by several researchers and has contributed to the development not only of NF-κB signaling but also other signaling research as well.^75,76)^

**Figure 12.**
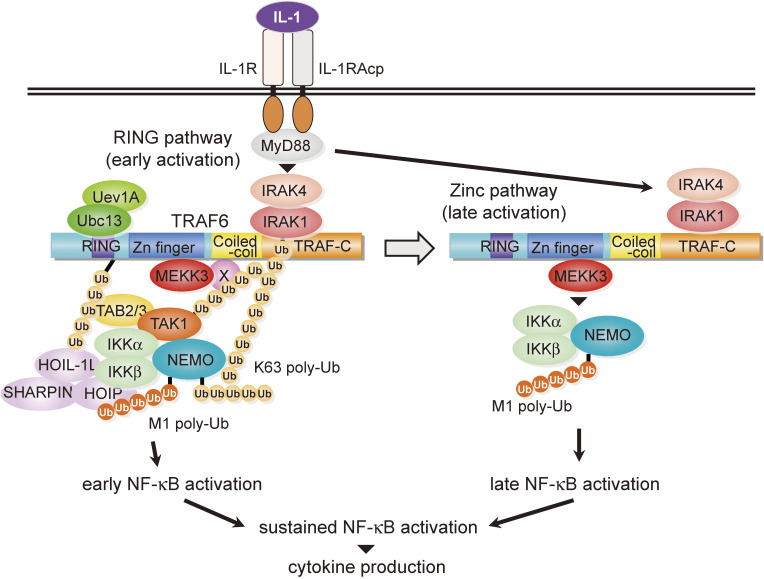
A model illustrating TRAF6-mediated IL-1 receptor signaling. In this model, IL-1R signaling consists of two mechanistically and temporally distinct pathways: the RING pathway and the Zinc pathway. The RING pathway transduces signals by the TRAF6/MEKK3/TAK1 complex. The binding of TAB2/3 to the K63-Ub chain conjugated to TRAF6, and the binding of MEKK3 or its associated protein (designated as X) to the K63-Ub chain conjugated to TAK1 is likely to stabilize the signaling complex. In addition, NEMO binding to the K63-Ub chain conjugated to TRAF6 or IRAK1 results in recruitment of the IKK complex to the signaling complex. TRAF6 catalyzes these polyubiquitin reactions. Such ubiquitin chain mediated protein complex formation may trigger the MEKK3-TAK1-IKK kinase cascade, thereby leading to NF-κB activation. The Zinc pathway is independent of TAK1 but dependent on MEKK3, and the pathway is preceded by the RING pathway during the first hour after stimulation with IL-1. Cooperation of the two distinct pathways is required to produce sufficient amounts of inflammatory cytokines. The M1-Ub chain conjugated to NEMO is also involved in IKK activation.

Based on the Chen model, we further analyzed IL-1 signaling. IL-1 plays an important role in immune responses such as inflammatory reactions and infection defense,^77)^ and its unregulated expression is known to contribute to the malignant transformation of cancer.^78,79)^ Therefore, elucidation of the signaling mechanism of IL-1 will greatly contribute to the development of therapeutic agents for immune diseases and cancer. Binding of IL-1 to the IL-1 receptor induces intracellular signaling to activate NF-κB, resulting in cytokine and chemokine expression. In this signal transduction, TRAF6 polyubiquitinates itself with K63-Ub chains, and the TAK1/TAB2 complex binds to TRAF6 through this polyubiquitin chain. On the other hand, MEKK3 is also involved in IL-1 signaling as well as TAK1.^80,81)^ However, although TAK1, MEKK3, and TRAF6 are deeply involved in IL-1 signaling, the molecular mechanism of their activation had remained unclear. We focused on the E3 activity of TRAF6 and found that, upon IL-1 stimulation, TAK1 undergoes K63-type polyubiquitination catalyzed by TRAF6 (E3) and Ubc13 (E2), which is required for TAK1 activation.^82)^ We also found that MEKK3 is required for TAK1 activation and that TRAF6, TAK1, and MEKK3 form a signal complex in an IL-1 signaling-dependent manner. Because this complex was not formed in the absence of TAK1 polyubiquitination, a new signaling regulatory mechanism was revealed in which TAK1 itself undergoes K63-type polyubiquitination to promote signal complex formation to activate itself. Although the pathway mentioned above mediated by TAK1 polyubiquitination requires the RING domain of TRAF6 (RING pathway), we have shown previously an NF-κB activation pathway emanating from the Zinc domain of TRAF6 (Zinc pathway).^28)^ We then analyzed the Zinc pathway in detail and clarified that the Zinc pathway activates NF-κB with a temporal delay compared with the RING pathway. Therefore, we proposed that the Zinc pathway is mechanistically and temporally different from the RING pathway in terms of NF-κB activation. Furthermore, among the NF-κB target genes, there are groups whose expression is highly dependent on the Zinc pathway (TNFα, CCL2, CXCL10) and groups whose expression is less dependent on the Zinc pathway (IL-6, IRF1), thus clarifying the physiological role of the Zinc pathway. Based on the above results, we proposed a biphasic model of NF-κB activation by TRAF6, in which TRAF6 plays a role as a conductor that cleverly manipulates downstream molecules to precisely control inflammatory reactions and immune responses induced by IL-1 signaling (Fig. 12).^82)^

## Future perspective

In this review, we have comprehensively described the physiological role of TRAF6, its role in disease pathogenesis, and the molecular mechanisms by which TRAF6 activates downstream signaling, including NF-κB activation. Here, we describe the future challenges and prospects for TRAF6 research.

### 1 Cooperation of the distinct types of ubiquitin chains.

As mentioned above, TRAF6 research has led to a breakthrough in the signal transduction field. In addition to the classical role of ubiquitin in proteolysis, K63-Ub chains act as a scaffold for the formation of the signal complex.^75,76)^ Subsequently, M1-Ub chains (also called linear ubiquitin chains) were identified.^83–85)^ M1-Ub chains are synthesized by the E3 linear ubiquitin chain assembly complex (LUBAC) and were reported to be involved in the activation of the IKK complex leading to NF-κB activation in TNFR and IL-1 receptor signaling.^86,87)^ TRAF6 is not required for TNFR signaling, but it is crucial for IL-1R signaling. In the case of IL-1R signaling, it has been reported that a hybrid-type chain consisting of M1- and K63-Ub chains covalently linked to each other (M1/K63-Ub hybrid chains) are synthesized for IKK complex activation.^88)^ We have reported that M1/K63-Ub hybrid chains were also involved in NF-κB activation by Tax, an oncogenic protein of human T-cell leukemia virus type 1, which is a causative agent of adult T cell leukemia.^89)^ Although the conformations of M1 and K63 ubiquitin chains are similarly linear, the spatial configuration of the individual ubiquitin molecule in the chain is slightly different. It is not clear why M1/K63-Ub hybrid chains are required, but it may have a significant advantage in activating the signal complex. In addition to M1/K63-Ub hybrid chains, it has been reported that K48-K63 branched chains regulate IL-1-induced NF-κB activation.^90)^ In addition to these chains, there could be K6-, K11-, K27-, K29-, and K33-Ub chains, depending on the position of the lysine residue of the proximal ubiquitin to which the C-terminus of the distal ubiquitin attaches. The involvement of these ubiquitin chains and mixed chains with other combinations in biological phenomena, in which TRAF6 are involved, will be elucidated in detail in the future.

### 2 Tuning of TRAF6 signaling by its upstream receptor.

Osteoclastic progenitor cells express RANK and CD40, both of which are members of TNFRSF. When RANK is stimulated with RANKL, NF-κB activation is followed by sustained expression and activation of NFATc1, leading to osteoclast differentiation. However, when CD40 is stimulated with CD40L in osteoclastic progenitor cells, CD40 signaling activates NF-κB in a TRAF6-dependent manner, but NF-κB activation does not lead to osteoclastogenesis.^29)^ RANK-induced NF-κB activation persists for more than 24 hours, whereas CD40-induced NF-κB activation persists for less than 6 hours, thereby activating NFATc1 only transiently, indicating that transient activation of TRAF6 is not sufficient for osteoclast differentiation and that RANK can sustain the signal by some mechanism not present in CD40. By generating deletion mutants of RANK and RANK/CD40 chimeric receptors, we found that the cytoplasmic region of RANK, which we named the highly conserved region in RANK (HCR), contributes to signal persistence.^91)^ This result suggests that the HCR itself or a protein that binds to the HCR acts directly or indirectly on TRAF6 to sustain TRAF6 activation. Because such receptor-mediated tuning of TRAF6 signaling is essential for cell differentiation and its mechanism remains poorly understood, we are trying to elucidate the molecular mechanism to establish the concept that the TNFRSF (or even other types of receptors) can temporally tune the intensity and duration of the signal emanating from the downstream signal transducer to their purpose.

### 3 TRAF6-targeted drug discovery.

Based on an analysis of TRAF6-KO mice, if we can develop a TRAF6-specific drug, it could be used as an anti-inflammatory drug to suppress the cytokine storm, a drug for osteoporosis, and a drug for breast cancer. Because the enzymatic activity of TRAF6 is dependent on the RING structure,^75)^ we are trying to develop compounds that inhibit RING-dependent enzymatic activity. In collaboration with Fujita’s group in Kumamoto University, we found the chemical compound SN-1 inhibits RING function by withdrawing Zn ion from the Zinc finger near the RING.^92)^ The compound can be used as a lead compound to increase the specificity of TRAF6. We believe that it is necessary to continue our research in the hope that basic research on TRAF6 will lead to drug discovery and contribute to humanity’s welfare.

## Figures and Tables

**Figure 3.**
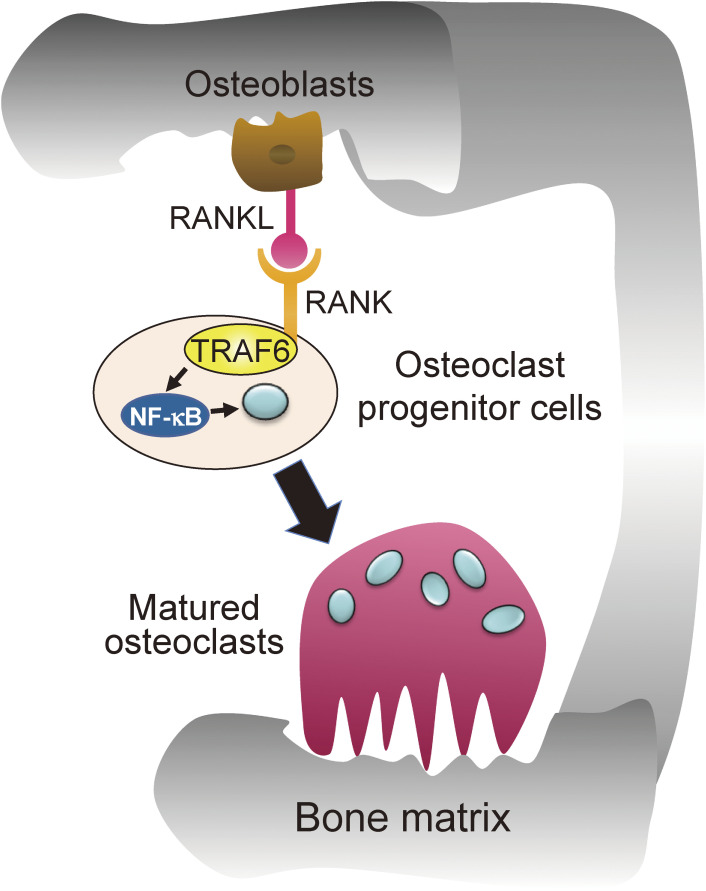
RANK-TRAF6 signal in osteoclast progenitor cells promotes osteoclastogenesis. In bone tissue, upon binding of RANKL expressed in osteoblasts to RANK on osteoclast precursor cells, TRAF6 is recruited to the cytoplasmic tail of RANK, leading to the activation of NF-κB and AP-1. In cooperation with the signal from the immunoreceptor tyrosine-based activation motif (ITAM)-harboring adaptors to activate calcineurin, TRAF6 signal activates NFATc1, a master transcription factor of osteoclastogenesis, to induce the formation of multinucleated mature osteoclasts.

**Figure 4.**
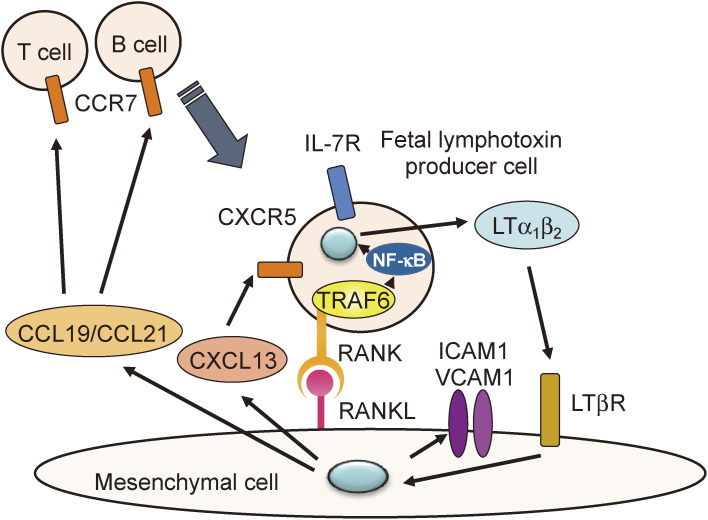
RANK-TRAF6 signal in IL-7Rα^+^ fetal lymphotoxin producer cells promotes lymph-node organogenesis. IL-7Rα^+^RANK^+^CXCR5^+^ fetal lymphotoxin producer cells are recruited to neonatal lymph node anlagen due to CXCL13 expression in lymphoid organ-specific LTβR^+^ mesenchymal cells. RANKL expressed in mesenchymal cells stimulates RANK in the fetal lymphotoxin producer cells. RANK activates the TRAF6-NF-κB pathway to induce secretion of LTα_1_β_2_. LTα_1_β_2_ then stimulates LTβR in mesenchymal cells to induce the secretion of adhesion molecules (ICAM1 and VCAM1), cytokines, and chemokines (CCL19/21). These chemokines recruit CCR7^+^ T and B lymphocytes to the neonatal lymph node anlagen leading to lymph node formation.

**Figure 5.**
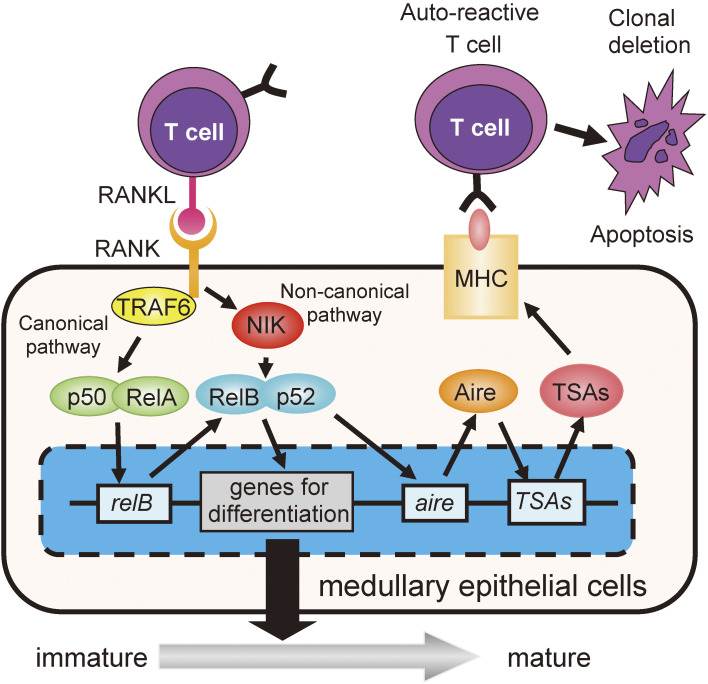
RANK-TRAF6 signal in medullary epithelial progenitor cells promotes maturation of medullary epithelial cells. RANKL expressed in T cells stimulates RANK on progenitors of medullary epithelial cells (mTECs). TRAF6 then activates the canonical NF-κB (p50/RelA) pathway to induce RelB expression. In cooperation with the RelB induction, RANK also activates the non-canonical NF-κB (p52/RelB) pathway, which in turn induces the expression of genes involved in the development and maturation of mTECs. During maturation, the RANK-mediated activation of the non-canonical NF-κB induces Aire expression. Aire then induces the promiscuous gene expression of TSAs. These autoantigens are presented with MHCs to auto-reactive T cells to induce their apoptosis, thereby establishing negative selection of T cells.

**Figure 6.**
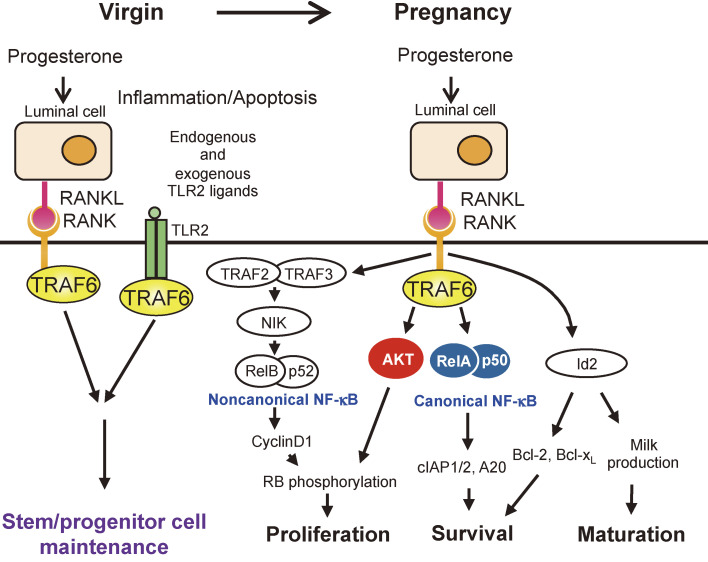
RANK-TRAF6 signal in mammary epithelial cells maintains mammary stem cells and promotes pregnancy-induced mammary epithelial cell expansion. Progesterone induces RANKL expression in luminal epithelial cells. RANKL then stimulates RANK in both luminal and basal epithelial cells. The TRAF6 pathway maintains mammary stem cells and luminal progenitor cells prior to pregnancy. During pregnancy, TRAF6-induced canonical NF-κB activation is involved in the survival of epithelial cells, whereas TRAF6-induced AKT activation is involved in the proliferation of epithelial cells.

**Figure 7.**
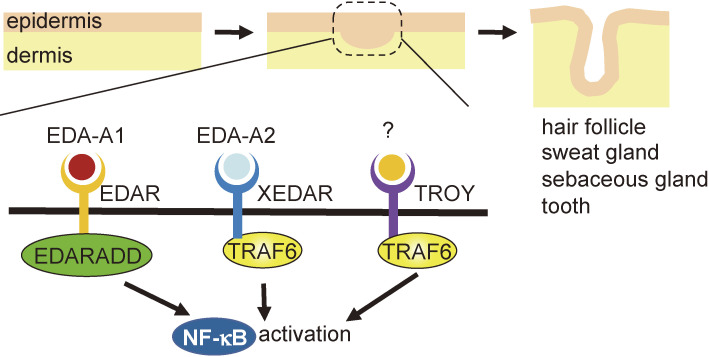
TRAF6 is required for skin appendix formation. EDAR, XEDAR, and TROY belong to TNFRSF and are involved in the formation of skin appendices, including hair follicles, sweat glands, sebaceous glands, and teeth. TRAF6 is involved in NF-κB activation by XEDAR and TROY.

**Figure 10.**
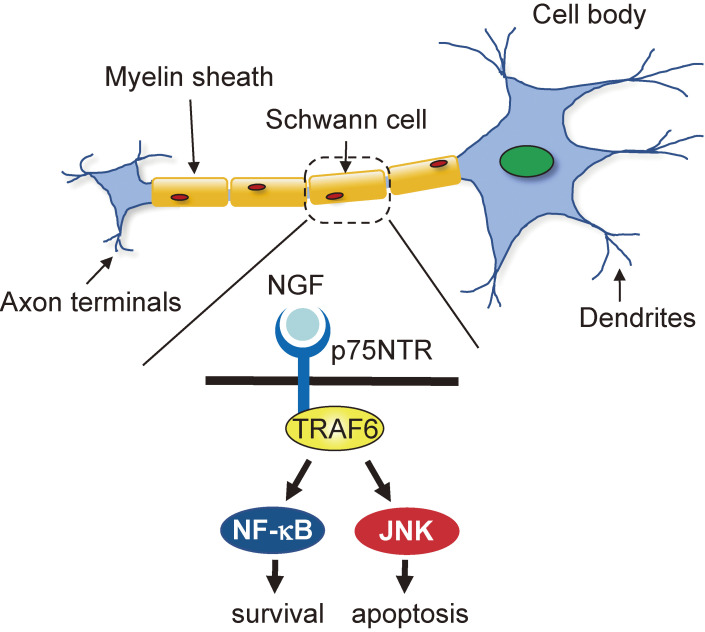
TRAF6 is crucial in p75 neurotrophin receptor signaling in Schwann cells. TRAF6 is an interactor of the p75 cytoplasmic tail. NGF activated NF-κB and JNK in WT Schwann cells, whereas their activation was significantly reduced in the absence of TRAF6.
